# Factors affecting the attitudes and opinions of ICU physicians regarding end-of-life decisions for their patients and themselves: A survey study from Turkey

**DOI:** 10.1371/journal.pone.0232743

**Published:** 2020-05-20

**Authors:** Nur Baykara, Tuğhan Utku, Volkan Alparslan, Mustafa Kemal Arslantaş, Nermin Ersoy

**Affiliations:** 1 Division of Critical Care, Department of Anesthesiology, School of Medicine, Kocaeli University, Kocaeli, Turkey; 2 Division of Critical Care, Department of Anesthesiology, School of Medicine, Yeditepe University, İstanbul, Turkey; 3 Division of Critical Care, Department of Anesthesiology, School of Medicine, Marmara University, İstanbul, Turkey; 4 Department of Medical History and Ethics, School of Medicine, Kocaeli University, Kocaeli, Turkey; Imam Abdulrahman Bin Faisal University, SAUDI ARABIA

## Abstract

**Introduction:**

Turkey is constitutionally secular with a Muslim majority. There is no legal basis for limiting life-support at the end-of-life (EOL) in Turkey. We aimed to investigate the opinions and attitudes of intensive care unit (ICU) physicians regarding EOL decisions, for both their patients and themselves, and to evaluate if the physicians’ demographic and professional variables predicted the attitudes of physicians toward EOL decisions.

**Methods:**

An online survey was distributed to national critical care societies’ members. Physicians’ opinions were sought concerning legalization of EOL decisions for terminally ill patients or by patient-request regardless of prognosis. Participants physicians’ views on who should make EOL decisions and when they should occur were determined. Participants were also asked if they would prefer cardiopulmonary resuscitation (CPR) and/or intubation/mechanical ventilation (MV) personally if they had terminal cancer.

**Results:**

A total of 613 physicians responded. Religious beliefs had no effect on the physicians’ acceptance of do-not-resuscitate (DNR) / do-not-intubate (DNI) orders for terminally ill patients, but atheism, was found to be an independent predictor of approval of DNR/DNI in cases of patient request (p<0.05). While medical experience (≥6 years in the ICU) was the independent predictor for the physicians’ approval of DNI decisions on patient demand, the volume of terminal patients in ICUs (between 10–50% per year) where they worked was an independent predictor of physicians’ approval of DNI for terminal patients. When asked to choose personal options in an EOL scenario (including full code, only DNR, only DNI, both DNR and DNI, and undecided), younger physicians (30–39 years) were more likely to prefer the "only DNR" option compared with physicians aged 40–49 years (p<0.05) for themselves and age 30–39 was an independent predictor of individual preference for "only DNR” at the hypothetical EOL. Physicians from an ICU with <10% terminally ill patients were less likely to prefer "DNR" or "DNR and DNI" options for themselves at EOL compared with physicians who worked in ICUs with a higher (>50%) terminally ill patient ratio (p<0.05).

**Conclusion:**

Most ICU physicians did not want legalization of DNR and DNI orders, based solely on patient request. Even if EOL decision-making were legal in Turkey, this attitude may conflict with patient autonomy. The proportion of terminally ill patients in the ICU appears to affect physicians’ attitudes to EOL decisions, both for their patients and by personal preference, an association which has not been previously reported.

## Introduction

End-of-life (EOL) decisions are a sensitive and challenging issue in terms of cultural, religious, and social aspects [1]. The EOL concept evolved in Western countries, and an ethicolegal framework has been established in most Western, developed countries [2,3]. However, data on EOL in other regions of the world, such as Asia, the Middle East, South America, and Africa, are relatively sparse [4,5].

It has been shown that country of origin, cultural background and religious background influence physicians’ attitudes and practice of medicine, including EOL care, although only in relatively few studies [6,7]. Some previous studies have shown that Muslim physicians are more likely than non-Muslim physicians to object to the concepts of withdrawal from life support or artificial nutrition, physician-assisted suicide, and terminal sedation [6, 8–11].

Turkey’s population is 98% Muslim (most are Sunni Muslim). However, Turkey has some specific features; Turkey is a secular country with a Muslim majority population and without a state religion. Although there has been one or two hours per week of compulsory religious courses in regular primary and secondary schools since 1982, the education system in universities, except for faculties of theology, is totally secular in Turkey. Turkey is a geographical and cultural bridge between Europe and the Middle East. Nevertheless, Islam has a considerable effect on cultural life, but the strength of this influence varies between the more and less developed regions of the country, between urban and rural populations, and between the social classes.

Due to medical advances, life expectancy has increased, and Turkey’s elderly population reached 8.5% of the entire population in 2017 [12]. Nearly 60% of deaths occur at hospitals in urban areas [13] and 88.2% of the population of Turkey resides in these urban areas [12].

Although the ICU bed counts in Turkey are comparable to those of countries in Western Europe [14], due to the very limited number of nursing homes, palliative care and insufficient home care and a lack of legal support to limit life support interventions for terminally ill patients, most Turkish ICUs are overcrowded [15]. A recent multicenter study showed that the average percent (±SD) of ICU bed occupancy 92.7% ±11.4% in Turkey [15].

The Turkish Medical Association issued an ethics declaration in 2008 about EOL care that would allow physicians to withhold or withdraw life support treatments in patients with terminal illnesses, emphasizing patient autonomy, and advised that when deciding to start a new treatment, the quality of life should be considered [16]. However, no specific law or standardized guidelines regulate EOL decisions in Turkey. The lack of legislation around EOL care places physicians in difficult situations in terms of EOL decisions. Thus, overtreatment at the EOL is often observed [17], which often is not in compliance with the patients’ wishes and creates a huge financial burden on the health system in Turkey, where the economy is not strong enough to support it.

There are very few studies on EOL issues in Turkey. İyilikçi et al. [18] investigated the practices of anesthesiologists in terms of EOL decisions in 2007. The present study was designed to identify the attitudes and opinions of ICU physicians regarding EOL decisions, for both their patients and themselves.

## Methods

The Kocaeli University Ethical Committee and Review Board approved the study (KÜ GOKAEK-2017/240). An online survey was distributed through SurveyMonkey, an online survey response collecting software.

The stages of the development and completion of the survey were as follows. The purpose of the survey was determined and, after a literature review of published studies from Turkey [18] and other countries [1–8], an expert group, including four intensivists, one statistician and one ethicist developed the survey. The target population was also defined as the members of national critical care societies of Turkey. Physicians who worked in pediatric and coronary ICUs were excluded. The time between survey delivery and collection of the responses was set at two months (stage 1). Following a pilot trial and minor revision (stage 2), the survey was distributed to the members of national critical care societies of Turkey (stage 3). Data file construction and analysis were performed (stage 4).

There was an explanation about the purpose and the use of the information gathered in the invitation e-mail sent to the ICU physicians. Participation by filling out the survey was assumed to imply consent; this assumption was approved by the ethics committee.

**Terminal illness** was defined as "An incurable and irreversible condition that would cause death within a reasonable period of time (in weeks or months) in accordance with accepted medical standards, and where the application of life-sustaining treatment would serve only to prolong the process of dying". Cases of brain death were not included in the study. The survey consisted of two parts and included 24 closed and 3 open-ended questions. Closed-ended questions included multiple-choice responses, but in some of them, there was also a category of "other" under which explanations could be given in free text format. The first part of the survey was designed to determine the physicians’ demographics, including age, sex, years of experience, religious beliefs, primary specialty, and ICU characteristics. The participant physicians’ opinion about the legalization of EOL decisions (including DNR/DNI orders), who should participate in EOL decisions, and when EOL discussions should be had, were determined in the second part of the survey. The participant physicians were also asked if they would prefer cardiopulmonary resuscitation (CPR) or intubation and mechanical ventilation (MV) for themselves if they were to be diagnosed with metastatic cancer that was unresponsive to treatment. The association of physicians’ sociodemographic and professional variables with their attitudes toward end-of-life decisions was investigated.

### Statistics

Statistical analyses were performed using SPSS version 20.0 software (SPSS Inc., Chicago, Illinois, USA). Questionnaires with at least 95% completed answers were included in the analysis. Descriptive statistics were applied to describe the sample’s professional and demographic characteristics as well as the physicians’ responses to the survey questions. Percentages were used to describe categorical data, and median (25-75th percentiles) was used to describe continuous data (age).

According to the expected and observed frequency, Pearson’s chi-square, Yates’ chi- square, or Fisher’s exact test was used for contingency tables, as required. Pearson’s chi-square or Monte Carlo simulation was applied for contingency tables that were larger than 2×2 according to the expected and observed frequency. Binary logistic regression analyses with backward stepwise selection were used to evaluate whether the physicians’ demographic and professional variables predicted the attitudes of physicians toward DNR (in cases of patient request) and DNI (for terminally ill patients and in cases of patient request) orders. Three separate binary logistic analyses were performed. For these analyses, undecided physicians were excluded, and only physicians who wanted or rejected the legalization of DNR/DNI orders were included in the binary logistic regression analyses. The three outcome variables were “Do you believe it is necessary to make changes to allow a DNR order in cases of patient request in Turkish criminal law?”, “Do you believe it is necessary to make changes to allow a DNI order for patients with terminal illness in Turkish criminal law?”, and “Do you believe it is necessary to make changes to allow a DNI order in cases of patient request in Turkish criminal law?”. As only 14 of the physicians opposed the legalization of DNR, the associations of DNR acceptance for terminally ill patients with physicians’ demographics and professional variables were not assessed statistically. For binary logistic analyses, the participants’ responses were converted into binary variables (Yes = 1 and No = 0). Variables with a p value of < 0.2 on univariate analysis were included in a multiple binary logistic regression analysis. Odds ratios (ORs) with 95% confidence intervals (CI) were reported.

Multinomial logistic regression analysis was used to evaluate whether the physicians’ demographic and professional variables predicted the physicians’ DNR/DNI preferences for themselves if they were to be diagnosed with metastatic cancer that was unresponsive to treatment. For the question, “If you had metastatic cancer that was unresponsive to treatment, would you prefer to forgo CPR and intubation /MV for yourself?”, the responses were: a.) Never (Group 1), b.) I am undecided (Group 2), c.) Yes, I would prefer only DNR (Group 3), d.) Yes, I would prefer only DNI (Group 4), e.) Yes, I would prefer both DNR and DNI (Group 5). Since only 4 physicians preferred the “only DNI” option, these four physicians were excluded from the multinomial logistic regression analysis. In multinomial logistic regression analysis, the first group consisted of the physicians who preferred “both DNR and DNI”, second group consisted of the physicians who preferred “only DNR” option. The reference category consisted of the physicians who did not have a positive attitude towards either DNR or DNI options for themselves (disagree or undecided). For this, physicians who did not prefer either DNR or DNI options (disagree) and physicians who were undecided were group into one variable, which constituted third group (reference group), due to small number of physicians in Group 1 and 2. The relationship between the ICU physicians’ individual DNR/DNI preferences and their demographic and professional variables were evaluated, and multinomial logistic regression models were constructed with a minimum of 10 outcome events per predictor variable. The model that best predicted the ICU physicians’ DNR/DNI preferences was determined. The relationships between physicians’ demographic, and professional variables and their opinion about patient and family participation in the decision process for DNR/DNI were also investigated by two separate binary logistic regression analyses.

Prior to building the models, multicollinearity was evaluated, with a variance inflation factor (VIF) of > 10 as an exclusion criterion. The VIF values of the selected variables were between 1.005 and 3.002. A *P* value of < 0.05 was considered significant.

## Results

Questionnaires were sent to 2004 ICU physicians and a total of 613 physicians responded to the survey (response rate of 30.5%). A total of 595 questionnaires with at least 95% completed answers were included in the analysis. The demographic characteristics of the participants are shown in Table 1. The median age of the ICU physician participants was 39 (33–45), and 54.5% were female. The primary specialty of the great majority of the ICU physicians was anesthesiology (88.2%), followed by internal medicine (9.9%) and surgery (1.9%). In terms of religious background, 504 (85.1%) of the participants considered themselves to be believers, 31 (5.2%) were indecisive, and 57 (9.6%) were atheists (Table 1).

**Table 1:** Characteristics of ICU physicians and their centers. Data presented as N (%) unless otherwise noted.

Characteristics	
**Age, yrs. (N = 595) Median (25–75%)**	39 (33–45)
**Age**	
30–39	315
40–49	191
>50	89
**Sex (N = 595) (F/M)**	324/271
**Religious affiliation (N = 592)**	
Believers	504 (85.1)
Undecided	31 (5.2)
Atheists	57 (9.6)
**Years of experience (N = 593)**	
≤2	185 (31.2)
3–5	150 (25.3)
6–10	119 (20.1)
>10	139 (23.4)
**Primary medical specialty (N = 591)**	
Anesthesiology	521 (88.2)
Internal medicine	59 (9.9)
Surgery	11 (1.9)
**Type of ICU (N = 590)**	
Mixed	526 (89.2)
Medical	38 (6.4)
Surgical	26 (4.4)
**Position (N = 593)**	
Attending	174 (29.3)
Staff	342 (57.7)
Resident/fellow	55 (9.3)
Others*	22 (3.7)
**ICU bed capacity (N = 594)**	
<10	127 (21.4)
11–20	281 (47.3)
>21	186 (31.3)
**The ratio of patients with terminal illness in the ICU**^a^ **(N = 592)**	
<10%	80 (13.5)
10–25%	193 (32.6)
25–50%	211 (35.6)
>50%	108 (18.2)
**Unavailability of ICU beds (N = 595)**	
Rare	11(1.8)
Sometimes	233(39.2)
Frequently	351(59.0)

The numbers may not add up to the total due to missing data.

* Physicians who work only night shift in the ICU.

^**a**^ Based-on data for the year preceding the survey, estimated annual percentage of terminally ill patients treated in the ICU.

A great majority of the ICU physicians (557; 93.6%) were of the opinion that it was necessary to make changes to allow DNR decisions for terminally ill patients in Turkish criminal law; 198 (33.3%) participants indicated that, regardless of patient prognosis, it was necessary to make changes to allow DNR decisions in all patients who do not want CPR, only 14 (2.3%) of the participants were opposed to the legalization of EOL decisions, and 23 (3.8%) were indecisive (Table 2). All 14 people who were against legalization of DNR were opposed because of humanitarian reasons; 2 were opposed for both humanitarian reasons and religious beliefs, and 2 were opposed for humanitarian reasons, religious beliefs and the threat of oppression and violence from relatives of patients.

**Table 2:** Physicians’ attitudes toward DNR/DNI orders.

Do you believe that it is necessary to make changes to allow DNR orders in the law?*	**N (%)**
a. Yes, for terminally ill patients	557 (93.6)
b. Yes, regardless of the prognosis, for all patients who do not want CPR.	198 (33.3)
c. Indecisive	23 (3.8)
d. Never	14 (2.3)
**Total response rate**	**595 (100)**
Do you believe that it is necessary to make changes to allow DNI orders in the law?*	**N (%)**
a. Yes, for terminally ill patients	426 (77.9)
b. Yes, regardless of the prognosis, for all patients who do not want intubation and invasive MV.	155(28.3)
c. Indecisive	50(9.1)
d. Never	55 (10.1)
**Total response rate**	**547 (100)**

* Percentages do not necessarily equal 100 because physicians may choose both a and b options.

DNR, do-not-resuscitate; DNI, do-not-intubate; CPR, cardiopulmonary resuscitation; MV, mechanical ventilation.

The proportion of participants who believed that legal changes should be made to allow DNR decisions in response to patient requests was higher among atheist physicians (49. 1%) than among believers (32.3%; p<0.05) and physicians indecisive on religion (22.1%; p<0.05). Having the viewpoint of atheism was an independent predictor of physicians’ approval of DNR decision in case of patient request when evaluated by multiple logistic regression analysis (S1 Table and Table 3).

**Table 3:** Factors affecting physicians’ attitudes toward DNR orders in cases of patient request.

	OR	95% CI	*P*
**Religious affiliation**			
Believers ^a^	1	-	
Indecisive	0.748	0.285–1.958	0.554
Atheists	2.004	1.066–3.769	**0.031**
**Gender**			
Female ^a^	1	-	
Male	1.464	0.986–2.173	0.059
**Unavailability of ICU beds**			
Rare ^a^	1	-	-
Sometimes	1.427	0.357–5.704	0.615
Frequently	0.876	0.215–3.569	0.854
**Type of ICU**			
Mixed ^a^	1	-	
Medical	0.432	0.095–1.975	0.279
Surgical	1.440	0.582–3.562	0.430

Model *χ*^2^ = 18.659, *P* = 0,009 –2 Log likelihood: 573.858

Hosmer Lemeshow *χ*^2^ = 5.490, *P* = 0.483.

^a^ Reference group.

*OR*, odds ratio; CI, confidence interval.

In total, 426 (77.9%) physicians wanted DNI to be legalized for terminally ill patients. (Table 2). An annual proportion of terminal patients admitted to the ICU where the physician worked that was between 10%–50% per year was an independent predictor for doctors to approve DNI for terminally ill patients (S2 Table and Table 4). 155 (28.3%) physicians stated that, regardless of the prognosis, legal changes should be made to allow DNI decision in cases of the patient request; 50 (9.1%) participants were indecisive, and 55 (10.1%) participants were opposed to the legalization of DNI (Table 2). The physicians’ religious beliefs (atheism) and the total duration of working years (≥6 yrs.) in the ICU were found to be independent predictors of the physicians’ support for legal changes to allow DNI orders in cases of patient request when evaluated by multiple logistic regression analysis (S3 Table and Table 5).

**Table 4:** Factors affecting physicians’ attitudes toward DNI orders in terminally ill patients.

	OR	95% CI	*P*
**The ratio of patients with terminal illness in the ICU**			
<10% ^a^	1	-	
10%-25%	3,140	1.436–6.865	**0.004**
25%-50%	2,351	1.130–4.893	**0.022**
>50%	1,765	0,769–4.051	0.180

* Based on data for the year preceding the survey, estimated annual percentage of terminally ill patients treated in the ICU.

Model *χ*^2^ = 8.221, *P* = 0,042 –2 Log likelihood: 381.273

Hosmer Lemeshow *χ*^2^ = 4.317, *P* = 0,827.

^a^ Reference group.

*OR*, odds ratio; CI, confidence interval.

**Table 5:** Factors affecting physicians’ attitudes toward DNI orders in cases of patient request.

	OR	95% CI	*P*
**Religious affiliation**			
Believers ^a^	1	-	
Indecisive	0.732	0.257–2.080	0.558
Atheists	2.239	1.266–4.287	**0.007**
**Years of experience**			
< 2 ^a^	1		
3–5	1.312	0.753–2287	0.337
6–10	1.831	1.048–3.200	**0.034**
>10	1.970	1.143–3.396	**0.015**
**Unavailability of ICU beds**			
Rare ^a^	1	-	
Sometimes	2.029	0.409–10.409	0.387
Frequently	1.249	0.247–6.308	0.788

Model *χ*^2^ = 21.226, *P* = 0,003; -2 Log likelihood: 585.741

Hosmer Lemeshow χ^2^ = 5.720, *P* = 0.573.

^a^ Reference group

*OR*, odds ratio; CI, confidence interval.

Responses to the question “If you had metastatic cancer unresponsive to treatment, would you prefer to forgo CPR and intubation /MV for yourself?” were received from 554 ICU physicians. A total of 373 (67.3%) of them expressed that they would choose to forgo CPR and intubation/mechanical ventilation, 106 (19.1%) would choose only DNR, 4 (0.7%) would choose only DNI, 29 (5.2%) would prefer no DNR/DNI, and 42 (7, 6%) were undecided (Table 6).

**Table 6:** ICU physicians’ preferences for themselves if they had metastatic cancer unresponsive to treatment.

**If you had metastatic cancer unresponsive to treatment, would you prefer to forgo CPR and intubation/mechanical ventilation for yourself?**	**N (%)**
a. No, I would prefer CPR and intubation/ MV, when required	29 (5.2%)
b. I am undecided	42 (7.6%)
c. I would prefer only DNR	106 (19.1%)
d. I would prefer only DNI	4(0.7%)
e. I would prefer both DNR and DNI	373(67.3%)
**Total response rate**	**554 (100%)**

CPR, cardiopulmonary resuscitation; DNR, do-not-resuscitate; DNI, do-not-intubate; MV, mechanical ventilation.

The DNR/DNI preferences of the ICU physicians for their own care, if they were to be diagnosed with metastatic cancer, were not changed by sex, religious beliefs, primary medical specialty or years of experience, when evaluated by chi- squared test (*P* >0.05). There were significant associations between the DNR/DNI preferences of the ICU physicians for their own care and age (*P* = 0.009), the annual proportion of terminally ill patients in the ICU (*P* = 0.014), and the frequency of unavailability of ICU beds in the ICU where the physicians worked, when evaluated by chi-squared test (*P* = 0.015). Compared with physicians who were between 40–49 yr old, physicians between 30–39 yr old more frequently stated that they would prefer the "only DNR" option (*P* <0.05) and age (30–39 yrs.) was an independent predictor of ICU physicians’ preference for the "only DNR " option, when evaluated by multinomial logistic regression (Table 7). According to the multinomial logistic regression analysis, physicians who worked in the ICU where the annual proportion of terminally ill patients less than 10% was less likely to prefer the "DNR" or "DNR and DNI " options for themselves at the end of life, compared with physicians who worked in an ICU which had a higher terminally ill patient ratio (> 50%) (Table 7).

**Table 7:** Factors affecting physicians’ DNR/DNI preferences for themselves. Results of Multinominal Logistic Regression Analysis.

ICU physicians’ preferences for themselves if they had metastatic cancer unresponsive to treatment		B	SE	Waid	Df	*P*	Exp (B)	95% Cl
**1**	**Intercep**	1.134	0.823	1.898	1	0,168		
	**Age, yrs.**							
	30–39	0.116	0.294	0.156	1	0.693	1.123	0.631–1.999
	> 50	0.211	0.414	0.259	1	0.611	1.235	0.548–2.781
	40–49	0^b^	.	.	0	.	.	.
	**The ratio of patients with terminal illness in the ICU** ^c^							
	<10%	-0.975	0.470	4.300	1	**0.038**	0.377	0.150–0.948
	25–50%	-0.116	0.436	0.071	1	0.790	0.890	0.379–2.092
	10-% 25%	-0.437	0.425	1.055	1	0.304	0.646	0.281–1.486
	>50%	0^b^	.	.	0	.	.	.
	**Unavailability of ICU beds**							
	Frequently	1.163	0.752	2.392	1	0.122	3.199	0.733–13.966
	Sometimes	0.445	0.749	0.353	1	0.553	1.560	0.359–6.775
	Rarely	0^b^	.	.	0	.	.	.
**2**	**Intercept**	-0.571	1.050	0.296	1	0.586		
	**Age, yrs.**							
	30–39	1.118	0.377	8.799	1	**0.003**	3.058	1.461–6.401
	> 50	0.855	0.517	2.729	1	0.099	2.351	0.853–6.481
	40–49	0^b^	.	.	0	.	.	.
	**The ratio of patients with terminal illness in the ICU** ^c^							
	<10%	-1.108	0.552	4.031	1	**0.045**	0.330	0.112–0.974
	10–25%	-0.678	0.499	1.847	1	0.174	0.507	0.191–1.350
	25–50%	-0.440	0.474	0.862	1	0.353	0.644	0.254–1.631
	>50%	0^b^	.	.	0	.	.	.
	**Unavailability of ICU beds**							
	**Frequently**	0.907	0.969	0.877	1	0.349	2.477	0.371–16.544
	**Sometimes**	0.654	0.967	0.457	1	0.499	1.923	0.289–12.801
	**Rarely**	0^b^	.	.	0	.	.	.

Model *χ*^2^ = 35.634, *P* = 0,001; -2 Log likelihood: 169.316

Pearson χ^2^ = 44.795, *P* = 0.605.

The reference category consists of physicians who did not have a positive attitude towards either the DNR or DNI options for themselves (disagree or undecided) (N = 72); 1. Physicians who prefer both DNR and DNI options (N = 369); 2. Physicians who preferred only DNR option (N = 105).

^*b*^ This parameter is set to zero because it was redundant.

^*c*^ Based on data for the year preceding the survey, estimated annual percentage of terminally ill patients treated in the ICU.

While 81.3% of the participant ICU physicians expressed that at least some of the life support treatment should be withheld, 18.6% of them expressed that no new treatment should be withheld in terminally ill patients (Table 8). A total of 76.7% of the ICU physicians expressed that at least some life support treatment should be withdrawn, and 23.1% of them expressed that none of the life support treatments should be withdrawn (Table 8). According to participant physicians, withholding inotropes/vasopressor agents was the most appropriate treatment-withholding method (58.2%), followed by MV (48.6%) and renal replacement treatment (RRT) (40.3%). 52% of the ICU physicians found withdrawing inotropes/vasopressor agents to be appropriate in terminally ill patients, followed by withdrawal of RRT (33.9%) and blood products (31.2%). For terminally ill patients, the rates of preference for withholding and withdrawal of any life-support treatment were similar (although the preference rates for withholding were higher than those for withdrawal), except for invasive MV. The preference for withholding invasive MV was 48.6%, whereas the preference for withdrawal was 22.7%. The withdrawing/withholding of fluid treatment, non-invasive mechanical ventilation (NIMV), and enteral nutrition were considered to be not appropriate by most of the participant ICU physicians (Table 8).

**Table 8:** Acceptance rates of ICU physicians for withholding or withdrawing of different treatments in terminally ill patients.

Treatment	Withholding N (%)	Withdrawing N (%)
**Inotropes/vasopressor**	322 (58.2)	288 (51.9)
**Invasive MV**	269 (48.6)	126 (22.7)
**RRT**	223 (40.3)	188 (33.9)
**Blood products**	183 (33.0)	173 (31.2)
**TPN**	132 (23.8)	119 (21.5)
**Antibiotics**	131 (23.7)	116 (20.9)
**Enteral nutrition**	76 (13.7)	57 (10.3)
**NIMV**	58 (10.5)	25 (4.5)
**Hydration**	43 (7.8)	25 (4.1)
**None of the any life sustaining treatments should be withheld/withdrawn**	103 (18.6)	128 (23.1)
**Total response rate**	553 (100)	554 (100)

*Percentages do not necessarily equal 100 because physicians might choose more than one treatment option.

MV, mechanical ventilation; RRT, renal replacement therapy; TPN, total parenteral nutrition;

NIMV, noninvasive mechanical ventilation.

For the question “Which of the patient related factors influence your DNR decision?”, 85.6% of the physicians indicated prognosis, followed by 50.7% who indicated quality of life, 49.8% who indicated the patient’s age, 43.0% who indicated comorbid illness, 35.5% who indicated patient/family request, 7.6% who indicated the unavailability of ICU beds, and 3.8% who indicated drug addiction.

For the question “Who should be involved in the decision process for DNR/DNI ?”, 529 (96.9%) of the physicians indicated the patient’s physician, followed by 379 (69.4%) who indicated the patient or their legal representatives, 273 (50.0%) indicated a consulting physician, 232 (42.5%) an ethics committee and 229 (41.9%) indicated family (Table 9).

**Table 9:** According to ICU physicians, the persons who should be involved in the decision process for DNR/DNI, in the case of a law change to allow EOL decisions.

	N (%)
**Physician**	529 (96.9)
**Patients or their legal representatives**	379 (69.4)
**Consulting physician**	273 (50.0)
**Ethics committee**	232 (42.5)
**Family**	229 (41.9)
**Total responders**	546 (100) *

* Percentages do not necessarily equal 100 because physicians might choose more than one answer.

DNR, Do-not-resuscitate; DNI, Do-not-intubate; EOL, End-of-life decisions.

Among physicians’ sociodemographic and professional variables, only age was independently associated with the opinion that patients or their legal representatives should be participated in the decision process for DNR/DNI, when evaluated by multivariate logistic regression analysis. Compared with younger physicians (<40 yrs), more physicians who were between 40–49 yr old (OR = 1.88, 95% CI = 1.228–2.901, *P* = 0.004) and > 50 yrs. old (OR = 1.98, 95% CI = 1.111–3.539, *P* = 0.021) were of the opinion that patients or their legal representatives should participate in the decision process for DNR/DNI in the case of a change in laws/regulations to allow EOL decisions.

Compared with physicians whose primary medical speciality was anesthesiology, more physicians whose primary medical speciality was internal medicine were of the opinion that a patient’s family should participate in EOL decisions, when evaluated by multivariate logistic regression analysis (OR = 2.08, 95% CI = 1.147–3.786; *P* = 0.016). Finally, more physicians whose total duration of working in the ICU was >2 years were of the opinion that the family of patients should participate in EOL decisions, than those with ≤2 years ICU experience. Compared with physicians with ≤2 years; for those with 3–5 years experience OR = 2.03, 95% CI = 1.253–3.293 (*P* = 0.004); for those with 6–10 years experience OR = 1.77, 95% CI = 1.067–2.947 (*P* = 0.027); and for those with >10 years experience OR = 2.54, 95% CI = 1.553–4.167 (*P*<0.001).

The question "Should patients with terminal illness be admitted to the ICU for acute health problems?" was also investigated. According to 189 (35.4%) of the 554 respondents, terminally ill patients should never be admitted to the ICU; instead, they should be treated on the wards or in palliative care units as far as possible. A total of 196 (35.7%) of the respondents stated that terminally ill patients should be admitted to the ICU for acute health problems, regardless of the expected life time, whereas 164 (29.8%) believed that the expected life time should be considered when deciding whether to admit terminally ill patients to the ICU. 53 (9.7%) of the respondents stated that the expected life time should be at least 3 months, 43 (7.8%) stated at least 6 months, 48 (8.7%) stated at least 9 months and 20 (3.6%) stated a least 12 months for terminally ill patients to be admitted to the ICU for newly developed acute health problems.

## Discussion

Turkey has made remarkable improvements in recent decades in terms of the health status of the population, the health resources available, and patient satisfaction [19]. However, ICUs are increasingly faced with the problem of admitting patients during the terminal period, and resources are usually not used wisely and effectively near death due to a lack of EOL policy.

This study evaluated a representative sample of Turkish ICU physicians, and a great majority of the ICU physicians expressed the opinion that it was necessary to make changes to allow EOL decisions in the Turkish criminal code. Although the DNI acceptance rate for terminally ill patient was lower (77.9%) than the DNR acceptance rate (94.1%), it was still quite high. However, only 33.3% and 28.3% of the physicians stated that DNR and DNI should be legalized, respectively, irrespective of patient prognosis, in cases of patient request. This may be due to a paternalistic approach in Turkish medicine. The physician-patient relationship in Turkey largely depends on the belief that a physician always does the best for their patient and should always protect life. However, having the viewpoint of atheism was found to be an independent predictors of physicians’ approval of the DNR/DNI decision in cases of patient request. Thus, it seems religious beliefs also play an important role. Even if legal changes were to be made in the future in Turkey that would permit the making of EOL decisions, this situation may pose a problem for patient autonomy, an important principle of modern ethics.

In this survey study, "How would you describe your religious beliefs?" was asked to the participant physicians and the answer options were "a.) believer, b.) undecided or c.) atheist." The survey did not ask respondents about their specific religion. In total 85.1% of the respondents described themselves as a believer. We can not be sure that all of these believer physicians were Muslim. However, due to the overwhelming majority of Turkey’s population (98%) being Muslim, it would not be wrong to think that at least most of them were Muslims.

According to Islamic doctrine saving life is encouraged and it is a sin for someone to end their own life; asking someone else to end your life for you is also considered an unforgivable sin. However, withholding and withdrawing treatment in terminally ill patients, which are more passive forms of euthanasia, have greater acceptability according to medieval Islamic texts [20,21]. Death is a natural process and is unavoidable for terminally ill patients. Brockopp claimed that passive euthanasia means allowing God’s plan to run its course [21]. Thus, physicians who defined themselves as believers may have found DNR and DNI decisions appropriate for patients who were terminally ill.

In previous studies, it was also shown that the physicians’ religious background affects their EOL decisions [6–7, 22–25]. Independent of specialty, doctors who defined themselves as nonreligious were more likely to make decisions that might shorten life, to give continuous sedation until death and to discuss these decisions with patients [22]. A previous US survey [23] showed that religious physicians tended to give less weight to the patient’s wishes compared to less religious physicians (47% vs 67%, respectively). The Ethicatt study [7] also showed that religious physicians and nurses wanted more extensive treatments than those who were not religious but were affiliated with a religion (Protestant, Catholic or Jewish), and a majority of Catholic (53%) and Jewish (66%) physicians would override patient’s wishes to forgo life-saving treatments, whereas a minority of Protestants would do so (35%). However, the findings on autonomy were due to regional differences, not the degree of religiosity, when evaluated by multiple logistic regression analysis [7].

The acceptance rate of DNR orders among Turkish ICU physicians was over 90%, which was one of the highest rates reported among Muslim physicians. In some previous studies performed in Muslim countries, different DNR acceptance rates for terminally ill patients were reported [26–29]. A new, small study [26] was performed among emergency room (ER) and ICU physicians in a tertiary care hospital in Saudi Arabia (which has a significant ratio of Western educated physicians) where DNR is allowed by a fatwa that allows life support machines to be withheld/withdrawn if the patient’s condition is hopeless; 95.5% of the participants stated that DNR was not against their religious beliefs. In another study conducted by Saeed et al. [1] in 2015, 461 Muslim physicians in the United States and other countries were studied; 66.8% of the participant physicians stated that DNR was not against their religious beliefs. A previous study [27] published in 2015 showed that 39% of Egyptian physicians rejected the concept of DNR, while another small survey [28] conducted in 2016 showed that 63% of the participant ICU physicians in a Palestinian study wanted DNR to be legalized, and 43.9% of the physicians in Kuwait stated that the Ministry of Health should legalized passive euthanasia under certain restricted conditions [29]. Previous studies show that Western-educated physicians or physicians who have opportunities for exposure to the West (rotations, conferences) [30], which is not rare among Turkish physicians, have higher acceptance rates of EOL decisions. The laic educational system in Turkey might also have a role in physicians’ higher acceptance rate of EOL decisions compared with other Islamic countries.

As mentioned earlier, there are no specific laws or standardized guidelines regulating EOL decisions in Turkey. There is also no existing legal precedent for EOL decisions in Turkey, to date. The lack of a specific law directly applicable to EOL care, means that in cases of lawsuits the adaptation of other non-specific laws to these cases is likely to give rise to a range of different legal views and, in all likelihood, confusion. This topic is currently a subject of debate among jurists in Turkey. However, there are some articles of law that can be associated with end-of-life decisions, in cases of lawsuits. To support suicide is a crime (Article 84) according to the Turkish Criminal Code, and one may be held liable for the death of a person when there is failure to perform a work-related responsibility (Article 83) [31]. Given this legal viewpoint, it can be inferred that a physician may be charged with not performing their duty to do all that is possible to save the life of a patient if a DNR/DNI order is given. According to the 13th article of the Regulation on Patients’ Rights [32] “Euthanasia is prohibited. The right to live cannot be relinquished for medical or other reasons. The life of a person can not be terminated by the consent of the person in question or anyone else”. Due to these legal fears and a lack of training on EOL care, Turkish physicians usually provide aggressive full support, regardless of prognosis [17].

In this study, we did not ask a question such as “Did you give any DNR/DNI orders?” due to similar legal concerns. However, in a previous survey performed among anesthesiologists in Turkey in 2004, 65.9% of the respondents indicated that they had given DNR orders, and 94.2% of the DNR orders were given verbally [18].

DNI orders entered into the clinical practice at about the same time as DNR orders, and usually DNI orders are accompanied by DNR orders [33]. Code status discussions mostly bundle cardiopulmonary resuscitation with intubation in countries where EOL decisions are legal. However, prearrest respiratory failure is a significant indication for intubation and MV is mostly used to treat prearrest causes of respiratory failure, such as acute respiratory distress syndrome (ARDS), pneumonia, chronic obstructive pulmonary disease (COPD), congestive heart failure (CHF), and postoperative respiratory failure. The mortality after in-hospital cardiac arrest is still >75%; however, the mortality after MV for isolated respiratory failure is <40% [34]. This situation could have caused the relatively lower rate of physicians’ acceptance rates of DNI orders compared with DNR orders.

Physicians have a crucial role in the EOL decision-making process. Physicians often initiate discussions about life-sustaining treatment, make recommendations about treatment plans, and educate patients and families. Ideally, the physicians’ approach should be solely dependent on the patient’s situation and preferences. However, in some previous studies, it was shown that sociodemographic and professional characteristics of physicians affect their attitudes to EOL decisions which brings a degree of subjectivity into these decisions [35–37]. In a previous study, it was shown that physician-related determinants often had a larger effect than patient-related opinions [35]. However, relatively few studies examined physician related factors influencing EOL decision-making. In those studies, in addition to religious belief, specialty such as oncology versus surgery, more experience, and place of training (especially Western education) were most frequently identified as physician-related factors affecting the EOL decision [35,37–39]. While the effect of gender of physician on EOL decision-making was less clear [36], race, region, country, age and personal experience with terminal illness of the respondent physicians were found to be the other factors that influenced the intensity of EOL care in some studies [35, 38–42]. According to a previous survey, physicians were more willing to withdraw life support if they were younger [42]. However, Alemayehu *et al* [41] showed that older physicians were less likely to choose intensive therapies in a scenario-based international survey of physicians. In the present study, age of physicians had no effect on their opinion related to EOL decisions for patients, but younger physicians (30–39 years) were more likely to prefer the "only DNR" option compared with physicians aged 40–49 years (*p*<0.05) for themselves. It was more likely that physicians who were older (>40 years) would hold the opinion that patients or their legal representatives should participate in EOL decisions compared with younger physicians. Religious beliefs (atheism; for acceptance of DNR/DNI decisions in cases of patient request), medical experience (working period in ICU ≥ 6 years; for acceptance of DNI decisions in cases of patient request) and the volume of terminally ill patients in the ICUs where they worked (between 10–50% per year; for acceptance of DNI decisions for terminally ill patients) were independent predictors of physicians’ approval of EOL decisions. This study contributes to the view that sociodemographic characteristics of physicians may affect EOL decisions. These results emphasize the need for interdisciplinary meetings and discussions before meeting the patient/family, and joint decision-making in order to increase objectivity of EOL decisions. The results of the study also suggest that, even if legal changes were made to allow EOL decisions in Turkey, it would also be necessary to give education and training to physicians in EOL care at the graduate and postgraduate level. A further point concerning the training of undergraduate and postgraduate medical students would be to stress patient autonomy in Turkey.

In the present study, 5.2% of the ICU physicians stated that they would not prefer either DNR and DNI for themselves when terminally ill, and 7.6% were undecided. 87.1% of the physicians expressed that they would choose to forgo CPR and/or intubation and MV for themselves when terminally ill. The DNR/DNI preferences of ICU physicians for themselves when considering scenario of metastatic cancer were not changed by sex, religious beliefs, primary medical specialty or years of experience. However; when evaluated by multinomial logistic regression analysis, physicians who worked in the ICU where annually proportion of terminally ill patients was less than 10% were less likely to prefer "DNR" or "DNR and DNI " options for themselves at the end of life, compared with physicians who worked in ICUs where there was a higher terminally ill patient ratio (> 50%). Compared with physicians who were between 40–49 yr old, physicians between 30–39 yr old more frequently stated that they would prefer only "DNR " option (p<0.05), and age (30–39 yr) was an independent predictor of ICU physicians’ preference for only "DNR " option. These findings suggest that physicians’ age and the volume of terminally ill patients in the ICUs where they work affect physicians’ attitudes toward EOL decisions for themselves. In a recent survey from US, 88.3% of the 1081 physicians who care seriously ill patients opted for "do-not-prolong" for themselves when terminally ill, whereas 11.7% opted for the full-code status for themselves [43].

Cultural attitudes toward telling the truth to patients and even family are still problematic in Turkey. In the present study, 30.5% of the ICU physicians believed that it is not necessary to involve the patient in EOL decision- making, although 69.5% believed patients should be involved. Almost 58% of the ICU physicians believed that it is not necessary to involve family in the EOL decision- making (only 42% of physicians believed that family should be involved). This ratio is surprisingly low, even for Turkey, and it is problematic. In Turkey, as a paternalistic society, communication efforts are generally made not by patients but by the next-of-kin. Moreover, in the ICU, patients may not be conscious, and surrogate decision-makers are mostly family members. In the previous study performed in 2004 among anesthesiologists in Turkey [18], in which most of the ICU physicians’ primary specialty was anesthesiology, more physicians (89.4%) preferred to involve family in the EOL decisions. One of the reasons for this decline might be rising violence against doctors in Turkey in recent years, which is mostly perpetrated by families of their patients [44]. However, in a recent study performed among 350 patients and relatives in Turkey, the participants wanted both themselves and their first-degree relatives included in the EOL decision process [45]. In previous studies, 70–80% of family members in the USA [46], 100% of family members in India [47], 79% of family members in Lebanon [48], and 44% of the family members [49] in France were involved in EOL decisions.

There are significant differences in terms of the most frequently withheld/withdrawn therapeutic interventions between regions, countries and even ICUs in the same country [17]. Ironically, decisions to withhold/withdraw life-sustaining treatment were found to be less frequent in low/lower-middle gross national income (GNI) countries than in high GNI countries [50]. In a previous study, withdrawing therapy was less common in Southern European countries, including Turkey, Greece, Israel, Italy, Portugal and Spain, than in northern European countries [6]. In the present study, the preference rates for withholding and withdrawing any life-support treatment are similar, although the preference rates for withholding rates was higher than that for withdrawing, with the exception of invasive MV.

However, the data obtained from this study show what physicians believe rather than what they currently do or will do if end-of-life decision making is legalized. The preference for withholding invasive MV was 48.6%, whereas the preference for withdrawing invasive MV was 22.7%, in the present study. Although there is no difference between withholding and withdrawing in terms of ethical principles, withdrawing is often seen as being more difficult and more active. In the present study, withdrawing/withholding vasopressor/inotropic agents and RRT were found to be among the most preferred withdrawal/withholding methods, whereas withdrawing/withholding fluid treatment, NIMV, and enteral nutrition were reported by most of the participant ICU physicians to not be appropriate. Food and drink symbolize compassion and care in Turkish culture, as in most other cultures [51]. In a previous study performed among pediatric intensive care nurses in Turkey, 68% agreed that artificial nutrition should be sustained at all cost [52]. Even though, according to Islamic bioethics, withholding and withdrawing life sustaining treatments are allowable in cases where the physicians are sure about the inevitability of death, basic nutrition should be sustained [53].

There are some limitations to our study. This is a survey, and the general limitations of survey-type studies are lack of flexibility and depth. The response rate of the survey was low (30.5%) which might have led to nonresponse bias. Low response rates were also reported in some previous survey studies on EOL (41–46.2%) [7–8,10,22]. A low response rate of online surveys has been reported by many researchers in recent years; the response rate for web surveys is estimated to be 11% lower than for surveys administered on paper [54]. Another issue, due to the lack of a legal basis for making EOL decisions in Turkey, was that some of the ICU physicians might have been unwilling to respond to the survey about this sensitive subject. As mentioned above, the data obtained from this study show what physicians believe rather than what they do or will do if end-of-life decision making is legalized. One of the other limitations of our study is that we did not determine the degree of religiosity of the physicians, which can affect end-of-life decision making [1,7]. In addition, data concerning the annual ratio of terminally ill patients in the ICU was based on recall, which could be potentially biased.

In conclusion, Turkish ICU physicians are under pressure due to a high ratio of terminally ill patients in their ICUs and an inadequacy of EOL policies. A great majority of the physicians surveyed agreed about the legalization of DNR and DNI orders for terminally ill patients, whereas most of the ICU physicians did not approve legal changes allowing DNR and DNI orders in patients who are not terminally ill but have requested DNR/DNI. The findings of this study suggest that the volume of terminally ill patients in the ICUs where they work affects physicians’ attitudes toward EOL decisions both for themselves and for their terminally ill patients.

## Supporting information

S1 Table Identification of the physicians’ socio-demographic factors associated with physicians’ acceptance of DNR in cases of patient request. (DOCX)

S2 Table Identification of the socio-demographic factors of physicians associated with physicians’ acceptance of DNI for terminally ill patients. (DOCX)

S3 Table Identification of the physicians’ socio-demographic factors associated with physicians’ acceptance of DNI in cases of patient request. (DOCX)

S1 Questionnaire (DOC)

S2 Questionnaire (DOC)
