# Supplementary material for: Factors affecting the attitudes and opinions of ICU physicians regarding end-of-life decisions for their patients and themselves: A survey study from Turkey
Source: PLoS One. 2020 May 20;15(5):e0232743. doi: 10.1371/journal.pone.0232743 (PMC7239490; doi:10.1371/journal.pone.0232743)
Supplement: S1 Table — (DOCX) [file pone.0232743.s001.docx]

**Supplemental Table 1: Identification of the physicians’ socio-demographic factors associated with physicians’ acceptance of DNR in cases of patient request.**

|  | **N** | **OR (95% CI)** | **P** |
| --- | --- | --- | --- |
| **Age** | 570 | 0.997 (0.975-1.017) | 0.689 |
| 30-39 | 302 | 1 |  |
| 40-49 | 182 | 0.816(0.553-1.206) | 0.816 |
| >50 | 86 | 0.935(0.566-1.544) | 0.793 |
| **Gender** |  |  |  |
| Female | 313 | 1 |  |
| Male | 253 | 1.367 (0.93, 1.858) | 0.078 |
| **Religious affiliation** |  |  | 0.034 |
| Believers | 481 | 1 |  |
| Indecisive | 28 | 0.656 (0.273, 1.576) | 0.346 |
| Atheists | 54 | 1.969 (1.118, 3.468) | 0.019 |
| **Years of experience** |  |  | 0.393 |
| <2 | 175 | 1 |  |
| 3-5 | 143 | 0.982 (0.613, 1.574) | 0.939 |
| 6-10 | 114 | 1.452 (0.891, 2.368) | 0.135 |
| >10 | 132 | 1.183 (0.736, 1.902) | 0.488 |
| **Primary medical specialty** |  |  | 0.191 |
| Anestesiology | 497 | 1 |  |
| Internal medicine | 59 | 0.780 (0.435, 1.398) | 0.404 |
| Surgery | 11 | 0.178 (0.023, 1.399) | 0.101 |
| **Type of ICU** |  |  | 0.121 |
| Mixed | 502 | 1 |  |
| Medical | 38 | 0.840(0.541, 2.172) | 0.819 |
| Surgical | 26 | 0.350(0.099, 1.231) | 0.056 |
| **ICU bed capacity** |  |  | 0.385 |
| <10 | 119 | 1 |  |
| 11-20 | 266 | 1.305 (0.825, 2.063) | 0.255 |
| >20 | 180 | 1.039 (0.633, 1.706) | 0.879 |
| **The ratio of patients with terminal illness in the ICU^a^** |  |  | 0.455 |
| <10 % | 71 | 1 |  |
| 10%-25 % | 190 | 1.195 (0.674, 2.118) | 0.542 |
| 25%-50 % | 200 | 0.880 (0.495, 1.565) | 0.663 |
| >50 % | 102 | 1.212 (0.643, 2.284) | 0.551 |
| **Unavailability of ICU beds** |  |  | 0.073 |
| Rare | 11 | 1 |  |
| Sometimes | 218 | 1.970 (0.243, 3.868) | 0.965 |
| Frequently | 338 | 1.477 (0.375, 5.812) | 0.577 |

***OR,*** univariate odds ratio; ***CI,*** confidence interval.

**^a^** Based on data for the year preceding the survey, estimated annual percentage of the terminally ill patients treated in the ICU.
