# Supplementary material for: Factors affecting the attitudes and opinions of ICU physicians regarding end-of-life decisions for their patients and themselves: A survey study from Turkey
Source: PLoS One. 2020 May 20;15(5):e0232743. doi: 10.1371/journal.pone.0232743 (PMC7239490; doi:10.1371/journal.pone.0232743)
Supplement: S2 Table — (DOCX) [file pone.0232743.s002.docx]

**Supplemental Table 2: Identification of the socio-demographic factors of physicians associated with physicians' acceptance of DNI for terminally ill patients.**

|  | **N** | **OR (95% CI)** | ***P*** |
| --- | --- | --- | --- |
| **Age** | 492 |  | 0.176 |
| 30-39 | 251 | 1 |  |
| 40-49 | 166 | 1.761(0.965-3.214) | 0.065 |
| >50 | 75 | 1.306(0.621-2.748) | 0.481 |
| **Gender** |  |  |  |
| Female | 269 | 1 |  |
| Male | 220 | 0.739 (0.442, 1.235) | 0.248 |
| **Religious affiliation** |  |  | 0.451 |
| Believers | 416 | 1 |  |
| Indecisive | 21 | 3.437 (0.453, 26.079) | 0.233 |
| Atheists | 49 | 1.231 (0.503, 3.018) | 0.649 |
| **Years of experience** |  |  | 0.946 |
| <2 | 143 | 1 |  |
| 3-5 | 124 | 0.847 (0.426, 1.683) | 0.635 |
| 6-10 | 103 | 0.974 (0.464, 2.046) | 0.945 |
| >10 | 117 | 1.042 (0.504, 2.153) | 0.912 |
| **Primary medical specialty** |  |  | 0.153 |
| Anesthesiology | 433 | 1 |  |
| Internal medicine | 49 | 4.151 (0.984, 17510) | 0.053 |
| Surgery | 7 | 1.104 (0.126, 8.948) | 0.957 |
| **Type of ICU** |  |  | 0.469 |
| Mixed | 436 | 1 |  |
| Medical | 31 | 0.000(0.000-) | 0.000 |
| Surgical | 21 | 0.000(0.000-) | 0.000 |
| **ICU bed capacity** |  |  | 0.321 |
| <10 | 105 | 1 |  |
| 11-20 | 226 | 1.628 (0.859, 3.085) | 0.135 |
| >20 | 156 | 1.420 (0.722, 2.795) | 0.310 |
| **The ratio of patients with terminal illness in the ICU^a^** |  |  | 0.051 |
| <10 % | 57 | 1 |  |
| 10%-25 % | 160 | 2.930 (1.325, 6.483) | 0.008 |
| 25%-50 % | 176 | 2.279 (1.076, 4.827) | 0.031 |
| >50 % | 94 | 1.715 (0.757,3.884) | 0.196 |
| **Unavailability of ICU beds** |  |  | 0.125 |
| Rare | 9 | 1 |  |
| Sometimes | 184 | 2.567 (0.608, 10.834) | 0.200 |
| Frequently | 295 | 3.639 (0.917, 16.059) | 0.065 |

***OR,*** univariate odds ratio; ***CI,*** confidence interval.

**^a^** Based on data for the year preceding the survey**,** estimated annually percentage

of terminally ill patients admitted to the ICU in a year.
