# Supplementary material for: Factors affecting the attitudes and opinions of ICU physicians regarding end-of-life decisions for their patients and themselves: A survey study from Turkey
Source: PLoS One. 2020 May 20;15(5):e0232743. doi: 10.1371/journal.pone.0232743 (PMC7239490; doi:10.1371/journal.pone.0232743)
Supplement: S3 Table — (DOCX) [file pone.0232743.s003.docx]

**Supplemental Table 3: Identification of the physician socio-demographic factors associated with physicians' acceptance of DNI in cases of patient request.**

|  | **N** | **OR (95% CI)** | **P** |
| --- | --- | --- | --- |
| **Age** | 492 |  | 0.835 |
| 30-39 | 251 | 1 |  |
| 40-49 | 166 | 0.929(0.606-1.424) | 0.735 |
| >50 | 75 | 1.109(0.640-1.921) | 0.712 |
| **Gender** |  |  |  |
| Female | 269 | 1 |  |
| Male | 220 | 1.170 (0.796, 1.719) | 0.424 |
| **Religious affiliation** |  |  | 0.039 |
| Believers | 416 | 1 |  |
| Indecisive | 21 | 0.714 (0.267, 2.077) | 0.573 |
| Atheists | 49 | 2.107 (1.157, 3.857) | 0.015 |
| **Years of experience** |  |  | 0.037 |
| <2 | 143 |  |  |
| 3-5 | 124 | 1.261 (0.728, 2.182) | 0.408 |
| 6-10 | 103 | 1.874 (1.076, 3.266) | 0.027 |
| >10 | 117 | 2.004 (1.173, 3.423) | 0.011 |
| **Primary medical specialty** |  |  | 0.636 |
| Anesthesiology | 433 | 1 |  |
| Internal medicine | 49 | 1.070 (0.570, 2.011) | 0.833 |
| Surgery | 7 | 0.368 (0.044, 3.086) | 0.357 |
| **Type of ICU** |  |  | 0.843 |
| Mixed | 436 | 1 |  |
| Medical | 31 | 0.727 (0.219, 2.412) | 0.603 |
| Surgical | 21 | 0.815(0.380, 1.749) | 0.600 |
| **ICU bed capacity** |  |  | 0.638 |
| <10 | 106 | 1 |  |
| 11-20 | 226 | 1.062 (0.646, 1.746) | 0.812 |
| >20 | 156 | 0.857 (0.500, 1.471) | 0.575 |
| **The ratio of patients with terminal illness in the ICU^a^** |  |  | 0.438 |
| <10 % | 57 | 1 |  |
| 10%-25 % | 160 | 0.991 (0.522, 1.882) | 0.977 |
| 25%-50 % | 176 | 0.729 (0.383, 1.388) | 0.335 |
| >50 % | 94 | 1.082 (0.540, 2.167) | 0.824 |
| **Unavailability of ICU beds** |  |  | 0.054 |
| Rare | 9 | 1 |  |
| Sometimes | 185 | 1.167 (0.234, 5.814) | 0.851 |
| Frequently | 295 | 1.906 (0.389, 9.341) | 0.427 |

***OR,*** univariate odds ratio; ***CI,*** confidence interval.

**^a^** Based on data for the year preceding the survey, estimated annual percentage of terminally ill patients treated in the ICU.
