# Supplementary material for: Factors affecting the attitudes and opinions of ICU physicians regarding end-of-life decisions for their patients and themselves: A survey study from Turkey
Source: PLoS One. 2020 May 20;15(5):e0232743. doi: 10.1371/journal.pone.0232743 (PMC7239490; doi:10.1371/journal.pone.0232743)
Supplement: S1 Questionnaire — (DOC) [file pone.0232743.s004.doc]

**Attitudes and opinions of ICU physicians in Turkey regarding**

**end-of-life decisions in 2017**

**Terminally illness** was defined as " An incurable and irreversible condition that would cause death within a reasonable period of time (in weeks or months) accordance with accepted medical standards, and where the application of life-sustaining treatment would serve only to prolong the process of dying" in this survey study. Cases of brain death should be excluded when answering the questions.

**1- Age:........................**

**2-Gender:** a. Female

b. Male

**3-How would you describe your religious beliefs?**

a.Believer

b. Indecisive

c. Atheist

**4- Your field of primary medical specialty...............................................**

**5-Year of becoming a specialist (primary medical specialty)**....................................................

**6- Do you have a super-speciality degree in critical care medicine?*****

a. Yes

b. No

* Until recently, ICUs were mainly managed by anaesthesiologists, followed by internists, pulmonologists, or surgeons, in Turkey. Critical Care Medicine has became a super-speciality with a multidisciplinary access recently in Turkey. Thus, only a small percentages of ICU physicians are certified in critical care medicine. However physicians whose primary speciality is anaesthesiology for which ICU rotation is mandatory during training, can still work in ICU and still constitute the majority of intensive care doctors in Turkey.

**7-What is your current position in the ICU?**

a.Attending

b.Staff

c.Resident/fellow

d.Others*

* Physicians who work only night shift in the ICU

**8-How long have you been working in the ICU?**

a. ≤ 2 yr

b. 3-5 yr

c. 6-10 yr

d. **>**10 yr

**9- What type of intensive care do you work in?**

a. Mixed

b. Medical

c. Surgical

**10-How many beds are there in the ICU in which you work?**

9. ≤ 10

b.11-20

c.>20

**11- In which level of ICU do you work?**

a. Level 1

b. Level 2

c. Level 3

**12-Do you ever refuse to admit patients in need due to full intensive care unit (ICU) bed occupancy?**

a.Often

b.Sometimes

c. Rarely

**13- What is the approximate rate of patient population admitted to the ICU within 1 year that are in the terminal period without any chance of recovery? ***

a. Less than 10%

b. Between 10-25%

c. Between 25-50%
d. More than 50%

***** Based on data for the year preceding the survey, estimated annual percentage of terminally ill patients treated in the ICU

**14- Do you think that failure to make a do-not-resuscitate (DNR)**  **decision adversely affects the effective and rational use of ICU resources?**

a. Yes, the failure of a DNR decision is an important factor in the efficient and rational use of ICU beds

b. I’m not sure

c. No

**15- Do you believe that it is necessary to make changes to allow DNR orders in the laws and regulations? (More than one answer can be marked).**

a. Yes, for terminally ill patients.

b. Yes, regardless of the prognosis, for all patients who do not want CPR. The patient's decision should be respected if the patient does not wish to receive CPR in the event of cardiopulmonary arrest.

c. I’m not sure.

d. No, the decision for DNR should never be made . CPR should be applied to all patients experiencing cardiopulmonary arrest regardless of the prognosis.

**16- If your answer to question 15 is “no”, why? (More than one answer can be marked)**

a. I think life is very valuable. Everything necessary to keep a patient alive should be done independent of the prognosis.

b. Due to my religious beliefs

c. Due to the possibility of exposure to the pressure and violence of patient relatives

d. Other: Please specify: ……..

**17- If your answer to question 14 is “yes”, why? (More than one answer can be marked).**

a. Cardiopulmonary resuscitation leads to a more prolonged, more painful death period in cases of the terminal period, irreversible diseases . The result does not change; the patient suffers more.

b. Resources are not used rationally, no free bed is found for patients who need ICU care.

c. Even if the patient's prognosis is not poor, they should have the right to make decisions regarding their own body. The patient should be able to refuse cardiopulmonary resuscitation as well as other treatments.

d. Other: Please specify:..........

**18- In case of changes in laws/regulations that allow DNR, which of the following items will affect your decision?**

**(More than one answer can be marked).**

a. Prognosis

b. Patient’s age

c. Substance addiction of the patient

d. Comorbid diseases

e. Patient’s quality of life

f. Request of patient and/or their family

g. Need of free bed in ICU

h. Your religious belief

i. Other**.** Please specify:..........

**19- Should legal changes be made to allow the do-not-intubate (DNI) decision when respiratory failure develops requiring intubation and invasive mechanical ventilation? (More than one answer can be marked).**

a. Yes, for terminally ill patients.

b. Yes, regardless of the prognosis, for all patients who do not want intubation and invasive mechanical ventilation.

c. I’m not sure.

d. The decision for DNI should never be made; regardless of prognosis, intubation and invasive mechanical ventilation should be performed if necessary.

**20- Who should take part in the decision process for DNR/DNI in case of laws/regulations change that allow end-of-life decision? (More than one answer can be marked).**

a. Physician

b. Patient / surrogate decision maker

c. The relatives of the patient

d. Other specialists who were consulted

e. Medical Ethics Specialist

f. Other: Please specify............................................,

**21- In your opinion, when changes are made to laws/regulations to allow DNR/DNI, when should the patient be asked about their preferences?**

**More than one answer can be marked.**

a. These questions should never asked the patients, it is not humane.

b. All hospitalized patients should be asked whether they want DNR/DNI or not, regardless of the prognosis. Their answers should be signed.

c. Only patients in the terminal period should be asked whether they want DNR/DNI or not, and their answers should be signed.

d. In the period following the diagnosis of fatal disease, the patient should be asked whether they want DNR/DNI or not, before entering the terminal period.

d. Not only sick people, but also healthy people should be able to express and document their DNR/DNI preferences at any time in their lives.

**22- If you had metastatic cancer unresponsive to treatment, would you prefer to forgo CPR and intubation/mechanical ventilation for yourself**

a. No, I would prefer CPR and intubation/mechanical ventilation,

when required

b. I am undecided

c. I would prefer only DNI.

d. I would prefer only DNR

e. I would prefer both DNR and DNI

**23- Do you think that patients in the terminal period should be admitted to the ICU when they develop acute health problems?**

a. Yes, all patients have the right to benefit from ICU regardless of the prognosis

b. In the case when the supportive treatments to be applied to the patients in the ICU are successful, they should be admitted to the ICU if the expected life expectancy is at least 1 month.

c. In the case when the supportive treatments to be applied to the patients in the ICU are successful, they should be admitted to ICU if the expected life expectancy is at least 3 months.

d. In the case when the supportive treatments to be applied to the patients in the ICU are successful, they should be admitted to ICU if the expected life expectancy is at least 6 months.

e. ,In the case when the supportive treatments to be applied to the patients in the ICU are successful, they should be admitted to ICU if the expected life expectancy is at least 1 year.

f. These patients should be followed up in hospital wards or palliative care units, and they should not be admitted to the ICU due to acute conditions.

**24-** **In your opinion, which of the following life-sustaining therapies should not be initiated, even if needed in patients in the terminal period?**

**More than one answer can be marked.**

a. Inotropic/vasopressor agent

b.Total parenteral nutrition

c.Enteral nutrition

d.Antibiotics

e.Dialysis

f.Blood products

g. Non-invasive mechanical ventilation

h. Invasive mechanical ventilation

ı. Intravenous fluid therapy

j. None of the above life-sustaining treatments should be initiated

k. All treatments should be applied completely regardless of the prognosis.

**25- In your opinion, in the terminal period, which of the following life-sustaining treatments should be discontinued, even if needed? More than one answer can be marked.**

a. Inotropic/vasopressor agent

b. Total parenteral nutrition

c. Enteral nutrition

d. Antibiotics

e.Dialysis

f.Blood products

g. Non-invasive mechanical ventilation

h. Invasive mechanical ventilation

ı. Intravenous fluid therapy

i. Except analgesic/sedative agents, all treatments should be stopped.

j. None of the any treatment that has already been started should be stopped.

**26- Who should be involved in the decision-making process if any treatment that is being applied is to be discontinued, because the patient is in the terminal period, in the case of the laws/regulations allow?**

**More than one answer can be marked.**

a. Physician

b. Patient / surrogate decision maker

c. The relatives of the patient

d. Other specialists who were consulted

e. Medical Ethics Specialist

f. Other: Please specify............................................,

**27- Do you think course on ethics should be included in the critical care curriculum?**

a. Yes, ethics courses should definitely be included in the critical care curriculum

b. I’m not sure

c. I don't think there is such a need.
