# Supplementary material for: Factors affecting the attitudes and opinions of ICU physicians regarding end-of-life decisions for their patients and themselves: A survey study from Turkey
Source: PLoS One. 2020 May 20;15(5):e0232743. doi: 10.1371/journal.pone.0232743 (PMC7239490; doi:10.1371/journal.pone.0232743)
Supplement: S2 Questionnaire — (DOC) [file pone.0232743.s005.doc]

**2017’de Türkiye’deki Yoğun Bakım Doktorlarının Yaşam Sonu Kararları ile İlgili Görüş ve Tutumları**

**Bu anket çalışmasında ölümcül hastalık; kabul edilen tıbbi standartlara göre makul bir süre içinde (haftalar ya da aylar) ölüme neden olacak ve yaşam sürdürücü tedavinin uygulanmasının sadece ölüm sürecini uzatmaya hizmet edeceği, tedavi edilemez ve geri dönüşü olmayan durumlar olarak tanımlandı. Sorular cevaplandırılırken, beyin ölümü vakaları hariç tutulmalıdır.**

**1.Yaşınız:** .....................................

**2.Cinsiyet:** a. Kadın

b. Erkek

**3.Dini inanç açısından kendinizi nasıl tanımlarsınız?**

a.İnançlı

b.Kararsız

c.Ateist

**4.Primer uzmanlık alanınız (anadal) .............................................**

**5.Uzman olma yılınız?(anadal için)............................................**

**6.Yoğun bakım uzmanlığı setifikanız var mı?**

a.Evet

b.Hayır

**7.Yoğun bakımda çalışma pozisyonunuz?**

a. Sorumlu öğretim üyesi ya da şef

b.Uzman

c.Asistan/felow

d.Diğer*

*YBÜ'de nöbetlerde çalışan doktorlar

**8. Kaç senedir YBÜ'de çalışıyorsunuz?**

a. ≤2 yıl

b.3-5 yıl

c.6-10 yıl

d. >10 yıl

**9- Çalıştığınız YBÜ'nin tipi?**

a.Karışık

b.Dahili

c. Cerrahi

**10. Çalıştığınız YBÜ kaç yataklıdır?**

9. ≤10

b.11-20

c.>20

**11-Çalıştığınız YBÜ'nin seviyesi?**

a.1. seviye

b.2. seviye

c.3. seviye

**12- Yoğun Bakım yataklarının doluluğu yüzünden ihtiyacı olan hastaları kabul edemediğiniz oluyor mu?**

a.Sıklıkla

b.Bazen

c. Nadiren.

**13- YBÜ'nize 1 sene içinde yatan hasta popülasyonunun yaklaşık olarak kaçta kaçını iyileşme şansı olmayan, terminal dönemdeki oluşturuyor.**

a. % 10'dan az

b. % 10-25 arası

c. % 25-50 arası

d. % 50'den fazla.

* Önceki senenin verilerine dayanarak, yaklaşık senelik terminal dönem hasta oranı.

**14- DNR kararı alamamanın, yoğun bakım kaynaklarının etkin ve akılcı kullanılmasını olumsuz etkilediğini düşünüyor musunuz?**

a. Evet , YBÜ yataklarının etkin ve akılcı kullanılamamasında DNR kararı alınamaması önemli bir faktördür

b. Emin değilim

c. Hayır

**15- Sizce yasalarda ve yönetmeliklerde kardiyopulmoner arrest durumlarında kardiopulmoner resüsitasyon yapılmaması (DNR)’a izin veren değişiklikler yapılmalı mı?(Birden fazla şık işaretlenebilir).**

a. Evet, terminal dönem, iyileşme şansı olmayan hastalık durumlarında geçerli olmak üzere.

b. Evet, tüm hastalar için hastaların tedaviyi reddetme hakkı kardiyopulmoner resüsitasyonu da kapsayacak şekilde genişletilmeli. Prognozdan bağımsız olarak hastanın istememesi halinde kardiopulmoner arrest geliştiğinde KPR uygulanmamalı.

c. Kararsızım

d.Hayır. Kardiyopulmoner arrest geçiren tüm hastalara, prognozdan bağımsız olarak kardiopulmoner resüsitasyon uygulanmalı.

**16- 15. soruya cevabınız “hayır” şeklindeyse bunun sebebi? (Birden fazla şık işaretlenebilir)**

a. Bence yaşam çok değerlidir. Hastayı yaşatmak için gereken her şey prognozdan bağımsız olarak yapılmalı.

b. Dini inanışlarım gereği

c. Hasta yakınlarının baskı ve şiddetine maruz kalma ihtimali nedeniyle.

d. Diğer: Belirtiniz: ……..

**17- 15. soruya cevabınız “evet” şeklindeyse bunun sebebi? (birden fazla şık işaretleyebilirsiniz).**

a. Terminal dönem, geri dönüşümsüz hastalığı olanlarda kardiyopulmoner arrest durumlarında kardiopulmoner resüsitasyon uygulandığında ölüm süreci uzatılmış, daha ızdırap verici hale getirilmiş oluyor. Sonuç değişmiyor, hasta daha fazla acı çekiyor.

b. Kaynaklar akılcı kullanılmamış oluyor, gerçek YBÜ hastalarına yer bulunmuyor.

c. Hastanın prognozu kötü olmasa bile, kendi bedeniyle ilgili karar verme hakkı kendisinin olmalıdır. Hasta diğer tedavileri olduğu gibi kardiopulmoner resüsitasyonu da reddedebilmelidir.

d. Diğer: Belirtiniz............................................

**18- DNR’a izin veren yasa/yönetmelik değişiklikleri yapılması durumunda aşağıdaki maddelerden hangisi yada hangileri kararınızda etkili olur?**

(birden fazla şıkkı işaretleyebilirsiniz).

a. Prognoz

b. Hastanın yaşı

c. Hastanın madde bağımlılığı

d. Komorbid hastalıklar

e. Hastanın yaşam kalitesi

f. Hasta ve/ veya ailenin isteği

g. YBÜ’de yatak ihtiyacının olması

h. Dini inanışınız

i. Diğer: Belirtiniz............................................

**19-Entübasyon ve invaziv mekanik ventilasyon uygulaması gerektiren solunum yetmezliği geliştiğinde hastanın entübe edilmemesi (Do-not- intubate, DNI) kararı alınabilmesine izin veren yasal değişiklikler yapılmalı mı? Birden fazla şık işaretlenebilir.**

a. Evet, termina dönem/ geri dönüşümsüz hastalık durumlarında DNI kararı alınabilmeli, hastaya sadece non-invaziv yöntemlerle olabildiğince solunum desteği sağlanmalıdır.

b. Evet, hastanın entübasyon istememesi durumunda, prognozdan bağımsız olarak, hastanın kararına saygı duyulmalıdır. Gereksinim durumunda hastaya sadece non-invaziv yöntemlerle olabildiğince solunum desteği sağlanmalıdır.

c. Kararsızım

d. Asla DNI kararı alınmamalı, prognozdan bağımsız olarak,
gerekiyorsa entübasyon ve invaziv mekanik ventilasyon uygulanmalıdır.

**20. yaşam sonu kararlarına izin veren yasa/yönetmelik değişiklikleri yapılması durumunda DNR/DNI karar sürecine kimler katılmalı (birden fazla şıkkı işaretleyebilirsiniz).**

a. Hastayı takip eden doktoru

b. Hasta /yasal vasisi

c. Hasta yakını

d. Konsültasyon istenen diğer branş uzmanları

e. Tıp Etiği Uzmanı

f. Diğer: Belirtiniz............................................

**21. Sizce DNR/ DNI’a izin veren yasa/yönetmelik değişiklikleri yapılması durumunda hastaya bu konulardaki tercihleri ne zaman sorulmalı?**

Birden fazla şık işaretlenebilir.

a.Hiçbir zaman bu sorular hastaya sorulmamalı, insani değil.

b.Hastaneye yatan tüm hastalara prognozdan bağımsız olarak DNR/DNI konusundaki tercihleri yazılı olarak sorulup, imzalatılmalı.

c.Sadece terminal dönemdeki hastalara DNR/ DNI isteyip istemediği sorulmalı, cevapları imzalatılmalı.

d.Ölümcül hastalık teşhisi konduktan sonraki dönemde hasta terminal döneme girmeden önce DNR/DNI konusundaki tercihleri yazılı olarak sorulup, imzalatılmalı.

e.Sadece hasta insanlar değil, sağlıklı insanlar da hayatlarının herhangi bir döneminde DNR/DNI ile ilgili tercihlerini yapıp, belgelendirebilmeli.

**22- Arzu etmeyiz ama, tedaviye yanıtsız, metastatik Ca tanısı alsanız , kendiniz için DNR ya da DNI kararı almak ister misiniz ?**

1. Hayır. Gerektiği taktirde kardiopulmoner resusitasyon ve entübasyon/mekanik ventilasyon uygulanmasını tercih ederim.
2. Kararsızım
3. Sadece DNI kararı alırım.
4. Sadece DNR kararı alırım.
5. DNR ve DNI kararı alırım.
6. **23-Sizce terminal dönemdeki hastalar akut sağlık problemleri geliştiğinde yoğun bakım ünitesine kabul edilmeli mi?**
7. Evet, yoğun bakım ünitesinden yararlanmak prognozdan bağımsız olarak tüm hastaların hakkıdır.
8. Hastaya yoğun bakımda uygulanacak destek tedavileri başarılı olduğu takdirde, beklenen yaşam süresi en az 1 ay ise yoğun bakım ünitesine kabul edilmeli.
9. Hastaya yoğun bakımda uygulanacak destek tedavileri başarılı olduğu takdirde, beklenen yaşam süresi en az 3 ay ise yoğun bakım ünitesine kabul edilmeli.
10. Hastaya yoğun bakımda uygulanacak destek tedavileri başarılı olduğu takdirde, beklenen yaşam süresi en az 6 ay ise yoğun bakım ünitesine kabul edilmeli.
11. Hastaya yoğun bakımda uygulanacak destek tedavileri başarılı olduğu takdirde, beklenen yaşam süresi en az 1 sene ise yoğun bakım ünitesine kabul edilmeli.
12. Bu hastalar gelişen akut durumlarda hastane servislerinde, palyativ bakım ünitelerinde takip edilmeli, yoğun bakım ünitesine kabul edilmemelidir.

**24-Sizce, terminal dönemdeki hastalarda gereksinim duyulsa dahi aşağıdaki yaşam sürdürücü tedavilerden hangisi ya da hangilerinin başlanmaması uygun olur?**

Birden fazla şık işaretlenebilir.

a.İnotropik/vazopressor ajan

b.Total parenteral nutrisyon

c.Enteral nutrisyon

d.Antibiyotikler

e.Dializ

f.Kan ürünleri replasmanı

g. Non-invaziv mekanik ventilasyon

h.İnvaziv mekanik ventilasyon

ı. İntravenöz sıvı tedavisi

j.Yukarıdaki yaşam sürdürücü tedavilerin hiçbiri başlanmamalı

k.Prognozdan bağımsız olarak tüm tedaviler eksiksiz uygulanmalıdır.

**25- Sizce, terminal dönemdeki hastalarda, gereksinim olsa dahi, aşağıdaki yaşam sürdürücü tedavilerden hangisi ya da hangilerininin kesilmesi uygun olur? Birden fazla şık işaretlenebilir.**

a.İnotropik/vazopressör ajan

b.Total parenteral nutrisyon

c.Enteral nutrisyon

d.Antibiyotikler

e.Dializ

f.Kan ürünleri replasmanı

g. Non-invaziv mekanik ventilasyon

h.İnvaziv mekanik ventilasyon

ı. İntravenöz sıvı tedavisi

i. Analjezik/sedativ ajanlar dışındaki bütün tedavileri keserim.

j.Başlanmış olan hiçbir tedaviyi kesmem.

**26-Sizce yasa/yönetmeliklerin izin vermesi durumunda, uygulanmakta olan herhangi bir tedavi hastanın terminal dönemde oluşu nedeniyle kesilecekse, bu konuda karar sürecine kim ya da kimler katılmalıdır?**

Birden fazla şık işaretlenebilir.

a. Hastayı takip eden doktoru

b. Hasta /yasal vasisi

c. Hasta yakını

d. Konsültasyon istenen diğer branş uzmanları

e. Tıp Etiği Uzmanı

f. Diğer: .Belirtiniz...........................................

**27-Sizce yoğun bakım yandal eğitim müfredatına yoğun bakım etiği konuları dahil edilmeli mi?**

a.Evet, kesinlikle yoğun bakım etiği dersleri müfredata dahil edilmeli

b.Emin değilim

c.Böyle bir ihtiyaç olduğu kanısında değilim.
